# Fas Ligand enhances vessel maturation and inhibits vascular leakage associated with age-related macular degeneration

**DOI:** 10.21203/rs.3.rs-4331250/v1

**Published:** 2024-05-08

**Authors:** Adarsha Koirala, Ann Marshak-Rothstein, Bruce R. Ksander, Meredith Gregory-Ksander

**Affiliations:** 1 Schepens Eye Research Institute of Massachusetts Eye and Ear Infirmary, Department of Ophthalmology, Harvard Medical School, Boston, MA, USA; 2 Department of Medicine, UMass Chan Medical School, Worcester, MA, USA

**Keywords:** Choroidal neovascularization, vascular leakage, Fas ligand, vessel normalization, macrophage, age-related macular degeneration

## Abstract

Neovascular age-related macular degeneration (AMD), results from choroidal neovascularization (CNV), retinal edema and loss of photoreceptors. Previous studies suggested that Fas Ligand (FasL) on retinal pigment epithelial cells inhibited CNV by inducing apoptosis of infiltrating Fas+ vascular endothelial cells. However, induction of apoptosis depends on membrane-bound (mFasL) while the FasL cleavage product (sFasL) is neuroprotective. To better understand how FasL regulates the development of CNV, we used a mouse model of laser CNV to evaluate the development of CNV in mice with a FasL cleavage site mutation (ΔCS) and can only express the membrane-bound form of FasL. There was no significant difference in CNV size and area of vascular leakage in homozygous FasL^ΔCS/ΔCS^ mice when compared to wild type mice. Unexpectedly, heterozygous FasL^ΔCS/WT^ mice developed significantly less vascular leakage and showed accelerated neovessel maturation. However, CNV was not prevented in heterozygous FasL^ΔCS/WT^ mice if the Fas receptor was deleted in myeloid cells (FasL^ΔCS/+^ Fas^flox/flox^ Cre^LysM^). Thus, FasL-mediated CNV inhibition depends on the extent of FasL cleavage, and on FasL engagement of Fas+ myeloid cells. Moreover, accelerated neovessel maturation prevents vascular leakage in AMD.

## Introduction

Age-related macular degeneration (AMD) is an irreversible blinding disease that manifests as loss of central vision primarily in the older population (> 50 years of age)[1]. It is estimated that approximately 200 million people worldwide currently suffer from AMD, a number that is expected to increase to 288 million by 2040[2]. AMD occurs in two forms, dry and wet; the latter is the more severe form of the disease and leads to breakdown of the outer blood-retina-barrier leads to choroidal neovascularization (CNV) within the macula. The formation of these immature choroidal neovessels results in leakage of fluid and blood cells causing retinal edema and the ensuing death of photoreceptors. The current anti-vascular endothelial growth factor (VEGF) antibody therapy for neovascular AMD has been quite successful at preventing vascular leakage, resolving retinal edema, and preventing vision loss [3, 4]. However, this treatment requires long-term inhibition of VEGF by repeated intravitreal injections of anti-VEGF antibody, and if treatment is discontinued disease progression resumes. Since VEGF is also required to maintain normal retinal homeostasis and is secreted by many cell types within the retina such as retinal pigment epithelial cells (RPE), Müller cells, ganglion cells, vascular endothelial cells, and glial cells [5–7], the long-term inhibition of VEGF is predicted to have significant negative consequences such as impaired ciliary body and Müller cell function [7], two very important cell types required for maintaining a healthy retina. In addition, a significant number of wet AMD patients do not respond to anti-VEGF treatment while other patients who initially respond to treatment gradually become unresponsive after an extended treatment regime [8, 9]. Treatment associated complications such as hemorrhagic lesions [10] and outer retinal atrophy [3, 8, 11–13] have also been reported. Finally, the repeated intravitreal injections needed for successful treatment is a huge burden that is not tolerated well by patients resulting in frequent noncompliance, under dosing, and visual loss. For these reasons, there is a need for improved alternative approaches to treat neovascular AMD.

A considerable research effort has focused on the function of Fas/Fas ligand (FasL) interactions in the development and regulation of angiogenesis in the retina. FasL is a transmembrane protein expressed at low levels in retinal ganglion cells, RPE, vascular endothelial and Müller cells [14]. FasL is well known for triggering apoptosis in Fas receptor positive cells. The importance of FasL in controlling the growth and development of CNV was illustrated in FasL-defective (gld) mice as these mice displayed a significant increase in the severity of laser-induced CNV when compared to wild-type mice [15]. It has been proposed that FasL expressed by RPE cells triggers apoptosis of infiltrating Fas+ vascular endothelial cells, thus inhibiting angiogenesis and the subsequent development of CNV [14, 16, 17]. However, there is considerable evidence in other non-eye related model systems that FasL can also activate a variety of *nonapoptotic* pathways [18–20].

FasL exists as a membrane-bound protein (mFasL) or as a soluble fragment (sFasL) released by metalloproteinase cleavage of mFasL. FasL-induced apoptosis requires expression of the membrane form that can undergo extensive multimerization and then engage multimerized Fas that accumulates in lipid rafts[21, 22]; sFasL can prevent mFasL-induced apoptosis and also has non-apoptotic functions [23–30]. The relevance of FasL cleavage in CNV associated with AMD was previously investigated using systemic administration of doxycycline, a metalloproteinase (MMP) inhibitor, to inhibit cleavage of mFasL [31]. Doxycycline treated mice developed reduced CNV lesions in a laser-induced model of AMD. Reduced angiogenesis correlated with increased FasL expression on choroidal tissue and decreased sFasL in the serum and eye as measured by ELISA, consistent with the notion that blockade of mFasL cleavage led to increased expression of pro-apoptotic mFasL, and more effective elimination of Fas+ infiltrating vascular endothelial cells [15]. In apparent contrast to this premise, examination of surgically excised choroidal neovascular membranes from patients diagnosed with “wet” AMD did not find a correlation between the expression of mFasL on RPE cells and either the size of neovascular membranes, or apoptosis of choroidal endothelial cells [32, 33]. One important problem in the *in vivo* analysis of sFasL and mFasL function is the difficulty of specifically regulating FasL cleavage. While MMP inhibitors such as doxycycline can block proteolytic FasL cleavage, they also have a variety of other anti-inflammatory and anti-angiogenic effects that are independent of the Fas/FasL pathway [34]. Moreover, doxycycline may not block all FasL-cleaving enzymes.

Because of these issues, we decided to use a genetic approach that would allow us to specifically control the extent of mFasL cleavage. C57BL/6 mice with a gene-targeted mutation of the FasL metalloproteinase cleavage site (ΔCS mice) have previously been used to examine the role of mFasL and sFasL in murine glaucoma [35]. This mutation does not affect transcription or translation of FasL, or cell type specific expression of FasL. The difference between wild-type (FasL^WT/WT^) and FasL^ΔCS/ΔCS^ mice is simply that FasL^ΔCS/ΔCS^ mice express increased levels of mFasL and no sFasL [35]. Therefore, we expected that FasL^ΔCS/ΔCS^ mice would be protected from CNV. We also included FasL^ΔCS/WT^ heterozygous mice in our study, anticipating an intermediate phenotype.

Intriguingly, we found that expression of mFasL alone did not inhibit the development of CNV in a mouse model of laser induced CNV and therefore it is unlikely that CNV is limited by FasL-mediated apoptosis of invading neovessels. Rather, our data suggest that FasL can prevent vascular leakage via a *non*apoptotic pathway that promotes accelerated neovessel maturation. Through the use of LysM-Cre Fas^fl/fl^ mice, where Fas is not expressed by myeloid cells, we demonstrate that accelerated neovessel maturation depends on Fas signaling in myeloid cells and is highly dependent on the extent of FasL cleavage as only heterozygous FasL^ΔCS/WT^ mice, and not homozygous FasL^ΔCS/ΔCS^ mice, exhibited enhanced protection against laser-induced CNV. Importantly, these studies point to the induction of neovessel maturation as a potential new treatment strategy for the prevention of vascular leakage in “wet”-AMD.

## Results

### Membrane FasL alone does not inhibit laser-induced CNV

**Fig. 1:**
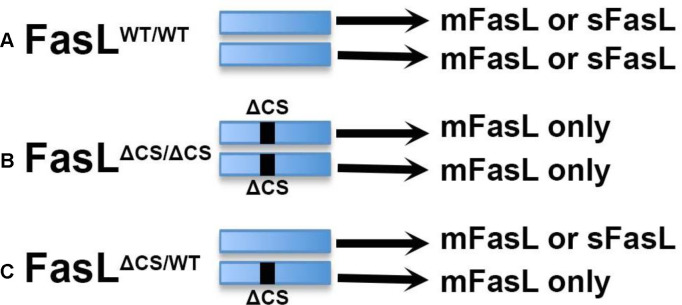
FasL alleles diagram. (A) Wild-type FasL^WT/WT^ mice in which both FasL alleles are wild-type and express normal levels of either mFasL or sFasL (normal mFasL and normal sFasL), (B) homozygous FasL^ΔCS/ΔCS^ mice in which both FasL alleles possess the ΔCS mutation (black line) and express greater than normal levels of mFasL and no sFasL and (C) heterozygous FasL^ΔCS/WT^ mice in which only one FasL allele possesses the ΔCS mutation and express greater than normal levels of mFasL and less than normal levels of sFasL as compared to WT mice (> normal mFasL and < normal sFasL).

**Fig. 2:**
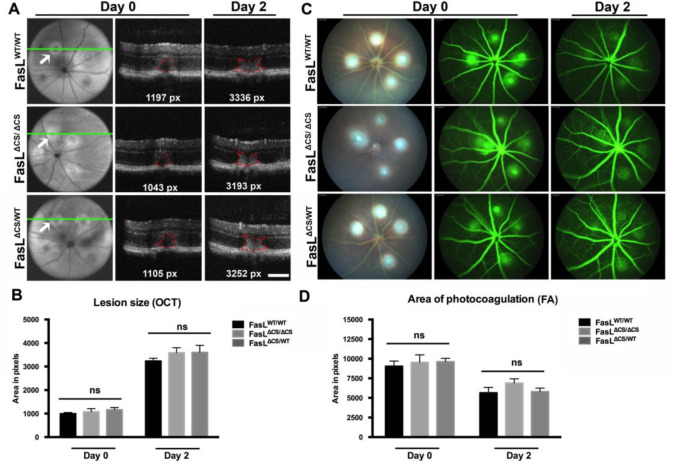
Evaluating consistency of laser-photocoagulation by SD-OCT and Fluorescein Angiography. (A) OCT images acquired at day 0 and 2 post laser. Left panel, a volume scan at day 0 post laser identifying the center of the lesion (green line) where the central hyporeflective area confirms a break in Bruch’s membrane. Middle panel, a rectangular b-scan taken through the center of the lesion (lesion identified by arrow) at day 0 shows the formation of the subretinal bubble immediately after laser injury (outlined in red, area in pixels). Right panel, a rectangular b-scan at day 2 shows the development of the characteristic butterfly-like lesion (outlined in red, area in pixels) (Scale bar = 0.1mm). Representative OCT images at Day 0 and Day 2 post laser from one animal in each group (N=10–16 per group). (B) Quantification of subretinal bubble and butterfly-like lesion was performed using OCT scans at day 0 and 2 post laser. Shown is the mean area in pixels ± SEM, 10–16 mice per group from 5 independent experiments (C) Fluorescein angiography performed at day 0 and 2 post laser. Left panel (day 0, bright field image), Middle panel (day 0, green filter). Right panel, (day-2, green filter). Representative fluorescein angiography images at Day 0 and Day 2 post laser from one animal in each group (N=10–16 mice per group). (D) Quantification of the area of photocoagulation at day 0 and day 2 post laser. Shown is the mean area in pixels ± SEM, 10–16 mice per group from 5 independent experiments. Statistical analysis was performed using 2-way ANOVA and Bonferroni’s multiple comparisons tests.

To distinguish the role of mFasL from sFasL in regulating CNV and vascular leakage, we used a 532 nm laser to induce CNV in three types of mice: (i) wild-type (WT) FasL^WT/WT^ mice that express normal levels of mFasL and sFasL, (ii) homozygous FasL^ΔCS/ΔCS^ mice that express more mFasL and no sFasL, and (iii) heterozygous FasL^ΔCS/WT^ mice that express greater than normal levels of mFasL and less than normal levels of sFasL (Figure 1). Laser photocoagulation of the retina produces an initial injury (days 0–2 post laser treatment) that is followed by the development of CNV (days 3–14 post laser treatment). We first determined whether the initial laser-induced injury observed on days 0–2 was affected by the FasL mutation in FasL^ΔCS/ΔCS^ mice. Using an image-guided laser system, we were able to precisely and reproducibly control location, size, and intensity of the laser photocoagulation required for CNV (Figure 2, Figure Supplement 1). As previously reported [36], laser treatment that successfully disrupts Bruch’s membrane produces a cavitation bubble that appears as a subretinal hyporeflective lesion on Day 0, as observed in lesions identified by fundus images (Figure 2A, far left column) and analyzed by SD-OCT (Figure 2A, middle column, red outline). Cavitation bubbles progress to a butterfly-like lesion on day 2 post laser treatment (Figure 2A, far right column, red outline). The size of retinal lesions on day 0 and 2 were not significantly different between WT, FasL^ΔCS/ΔCS^ and, FasL^ΔCS/WT^ mice (Figure 2B). Moreover, the area of vascular leakage as determined by fluorescein angiography on day 0 and 2 post laser treatment was also not significantly different between WT, FasL^ΔCS/ΔCS^ and, FasL^ΔCS/WT^ mice (Figure 2C middle and far right columns, respectively, and Figure 2D, ns p>0.05). We conclude that the partial (FasL^ΔCS/WT^) or complete FasL^ΔCS/ΔCS^) blockade of FasL cleavage did not affect the initial laser-induced injury within days 0–2, resulting in retinal lesions that were equal to those observed in WT mice at this initial time point.

**Fig. 3:**
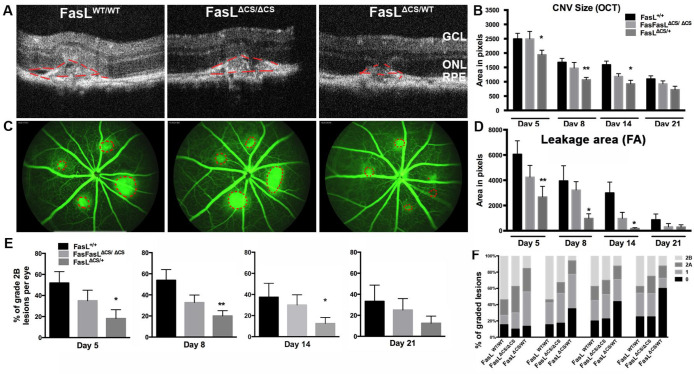
Expression of mFasL alone does not inhibit laser-induced CNV. (A) Representative OCT images acquired at day 5 post laser injury for FasL^WT/WT^, FasL ^ΔCS/ΔCS^, and FasL ^ΔCS/WT^ mice (Scale bar = 0.1mm), N=13–15 mice per group. (B) Quantification of CNV size (red outline) by SD-OCT at Day 5, 8, 14, and 21 post laser injury. Shown is the mean area in pixels ± SEM, N=13–15(D5), 13–14(D8), 9–12(D14), and 6–8(D21), from 5 independent experiments. (C) Representative FA images acquired at day 5 post laser injury for FasL^WT/WT^, FasL^ΔCS/ΔCS^, and FasL^ΔCS/WT^ mice, N=13–15 mice per group. (D) Quantification of the area of leakage (red outline) by FA at 5, 8, 14, and 21 days post laser injury. Shown is the mean area in pixels ± SEM, N =13–15 mice (D5), 13–14 mice (D8), 9–12 mice (D14), and 6–8 mice (D21), from 5 independent experiments. (E) CNV lesions were analyzed for active leakage using a previously established grading scheme [33]. The percent of 2B lesions (clinically significant leakage) was quantitated at Day 5, 8, 14, and 21 days post laser. Shown is the percent of grade 2B lesions per eye ± SEM, N=13–15 mice (D5), 13–14 mice (D8), 9–12 mice (D14), 6–8 mice (D21), from 5 independent experiments. (F) The percent of all lesions within each grade (0,1, 2A, and 2B) were quantitated at 5, 8, 14, and 21 days post laser in FasL^WT/WT^, FasL^ΔCS/ΔCS^, and FasL^ΔCS/WT^ mice. N=13–15 mice (D5), 13–14 mice (D8), 9–12 mice (D14), 6–8 mice (D21). P-values refer to 2-way ANOVA and Bonferroni’s multiple comparisons analysis: *P< 0.05, ** P< 0.01.

To examine CNV development in these three cohorts of mice, we then determined the size of CNV lesion by SD-OCT and the area of vascular leakage on days 5–21 post laser treatment. Vascular leakage was measured via fluorescein angiography at ~9–10 mins after i.p. injection of fluorescein (late phase). Consistent with a previous report [36], laser photocoagulation induced CNV in WT C57BL/6 mice began to develop on day 3, reaching a maximum on day-5 post laser treatment. However, SD-OCT revealed no significant differences in lesion size between WT and FasL^ΔCS/ΔCS^ mice at all days examined (days 5, 8, 14, and 21 post laser treatment) (Figure 3A and B). Fluorscein angiography revealed similar results, with no significant differences in the area of vascular leakage between WT and FasL^ΔCS/ΔCS^ mice at all days examined (Figure 3C and D). These data demonstrate that expression of higher-than-normal levels of mFasL alone prevented neither infiltration of choroidal vessels, nor the area of vascular leakage. Quite unexpectedly, the FasL^ΔCS/WT^ mice displayed a significant reduction in lesion size (Figure 3A and B) and vascular leakage area (Figure 3C and D) at day 5, 8, and 14 post laser treatment.

An alternative method to quantify vascular leakage uses fluorescein angiography (**FA**) and evaluates the increase in area and intensity of fluorescence at three time points: initial phase at ~1–2 min, middle phase at 4–5 min and late phase at ~ 9–10 min post fluorescein injection. The advantage of this technique is that it reflects active leakage from the lesions and, therefore, is believed to be a more clinically relevant assessment of vascular leakage [37, 38]. A previously established grading system was used to categorize the level of leakage as: none, minimal, moderate, and severe leakage (Grade 0, 1, 2A, and 2B, respectively) [37, 38]. As seen in the previous analysis, there was no significant difference between WT and FasL^ΔCS/ΔCS^ mice at any time point for grade 0, 1, 2A, and 2B lesions (Figure 3E and F). However, at days 5, 8, and 14 post laser treatment FasL^ΔCS/WT^ mice displayed significantly fewer grade 2B lesions (severe leakage) as compared with WT and FasL^ΔCS/ΔCS^ mice (Figure 3E and F). Thus, we detected a significant reduction in vascular leakage in FasL^ΔCS/WT^ mice, but not FasL^ΔCS/ΔCS^ mice, using two different methods. We conclude that FasL is capable of inhibiting both the size of CNV, as measured via SD-OCT, and the amount of vascular leakage, as measured by fluorescein angiography. However, this inhibition did not occur in homozygous FasL^ΔCS/ΔCS^ mice, where FasL cleavage was completely blocked, but only in heterozygous FasL^ΔCS/WT^ mice.

### Reduced vascular leakage in FasL^ΔCS/WT^ mice correlates with enhanced vascular maturity

**Fig. 4:**
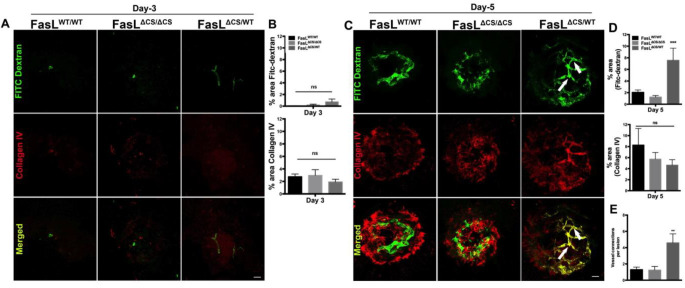
Analysis of neovessel structure. (A, C) Representative images from confocal analysis performed on FITC dextran (green) perfused RPE/Choroidal whole mounts stained for collagen IV (red) at Day 3 and Day 5 post laser (Scale bar =20μm) (40x magnification). (B, D) The area of FITC dextran staining (green) and area of collagen IV staining (red) was quantified in Z-stacked confocal images at (B) 3 days post laser (n=16–20) and (D) 5 days post laser (n=16–20 per group) Shown is the percent area ± SEM, N=10–16 mice per group, from 3 independent experiments. (E) The number of vascular intersections (white arrows) was quantified per lesion. Shown is the mean number of vessel connections per lesion ± SEM, N=10–16 mice per group, from 3 independent experiments. P-values refer to 2-way ANOVA and Bonferroni’s multiple comparisons analysis: **P<0.01, ***P< 0.001.

We expected the reduced leakage observed in the FasL^ΔCS/WT^ would be due to FasL-mediated inhibition of vessels growing into the lesion. To examine this, choroidal flat mounts were prepared from FITC-dextran perfused mice at 3 and 5 days post laser injury and stained for collagen IV (Figure 4). Collagen IV, a vascular basement membrane marker, was used to identify the CNV lesion [39, 40] while FITC-dextran was used to label all perfused vessels. Contrary to our prediction, there was no significant difference in collagen IV accumulation between the three groups of mice (WT, FasL^ΔCS/ΔCS^ and FasL^ΔCS/WT^) at either 3 or 5 days post laser injury (Figure 4A middle row, B, C middle row, and D). Moreover, there was an increase in perfused vessels growing into the lesions of FasL^ΔCS/WT^ mice. FITC-dextran perfused vessels began to appear in FasL^ΔCS/WT^ mice as early as 3 days post laser injury (Figure 4A top row and B) and by 5 days post laser injury there was a significant increase in the area of FITC-dextran perfused vessels in FasL^ΔCS/WT^ mice compared to WT and FasL^ΔCS /ΔCS^ mice (Figure 4C top row and D). Remarkably, perfused vessels in FasL^ΔCS/WT^ mice exhibited a more complex vascular network with increased branching as shown by a significant increase in vascular intersections as compared to the other groups (Figure 4C white arrows and E). Together, these data support the conclusion that the decreased vascular leakage observed in CNV lesions of FasL^ΔCS/WT^ mice is due to enhanced vascular maturation and/or vessel normalization.

### PDGFRb, TGFβ1 and pericyte co-localization with perfused vessels

**Fig. 5:**
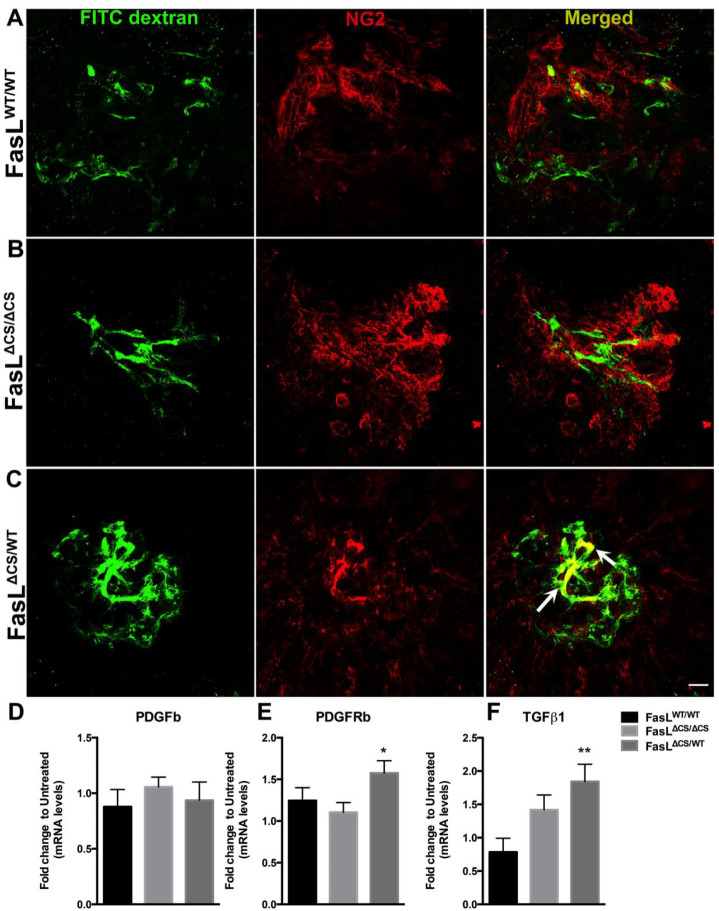
Co-localization of pericytes with perfused vessels and expression of PDGFb, PDGFRb, and TGFβ1. (A-C) Representative images from confocal analysis performed at 5 days post laser on FITC dextran (green) perfused RPE/Choroidal whole mounts stained for NG2 (red, pericytes marker) from FasL^WT/WT^ mice, FasL ^ΔCS/ ΔCS^ mice, and FasL ^ΔCS/WT^ mice. Merged images show co-localization of NG2 with FITC dextran perfused vessels (yellow, white arrows) in FasL ^ΔCS/WT^ but not FasL^WT/WT^ or FasL ^ΔCS/ ΔCS^ mice. (Scale bar-20μm) (40x magnification). Representative images from each group were taken from 2 independent experiments with N=8=10 per group (D-F) Quantitative PCR was performed on posterior eyecups at day-5 post laser to quantitate mRNA levels of PDGFb, PDGFbR, and TGF-β1. Data are shown as fold change from age-matched untreated controls ± SEM. N=8–10 mice per group from 3 independent experiments. P-values refer to one way ANOVA and Tukey’s multiple comparisons analysis, *P<0.05, **P<0.01.

Pericytes, which envelop newly forming vascular tubes, promote vessel normalization by inhibiting endothelial cell proliferation, migration, and production of VEGF-R2 [41, 42]. This process begins when sprouting endothelial cells release the chemotactic factor PDGFb (platelet derived growth factor beta) that in turn recruits PDGFRb (platelet derived growth factor Beta receptor) positive pericyte progenitor cells to the site of angiogenesis [43]. Endothelial cells also release TGFβ1 that induces pericyte progenitors to differentiate into mature pericytes [42]. Therefore, one mechanism to accelerate vessel normalization and reduce vascular leakage would be to increase pericyte co-localization of perfused vessels via increased recruitment and maturation of pericyte progenitors. To examine pericyte/vascular endothelial cell interaction directly within developing CNV lesions, we stained FITC dextran-perfused choroidal flat mounts from WT, FasL^ΔCS/WT^, and FasL^ΔCS/ΔCS^ mice at 5 days post laser injury for NG2, a pericyte marker. While NG2 staining was observed in CNV lesions from all groups of mice, only FasL^ΔCS/WT^ mice displayed significant co-localization of NG2 positive cells with FITC dextran perfused vessels (Figure 5A, B, and C). By contrast, the FasL^ΔCS/ΔCS^ mice displayed a similar NG2 staining pattern as WT mice, with no significant co-localization with FITC dextran perfused vessels (Figure 5A and B).

To determine if FasL affects recruitment and/or maturation of pericyte progenitor cells, we used quantitative real time PCR to determine mRNA levels of PDGFb, PDGFRb, and TGFβ1 in posterior eyecups (retina, RPE, choroid and sclera) isolated from WT, FasL^ΔCS/WT^, and FasL^ΔCS/ΔCS^ mice at 5 days post laser injury. No significant difference in PDGFb levels was observed between WT, FasL^ΔCS/WT^, and FasL^ΔCS/ΔCS^ mice (Figure 5D). By contrast, there was a significant increase in PDGFRb and TGFβ1 mRNA in FasL^ΔCS/WT^ versus WT mice, suggesting that FasL^ΔCS/WT^ mice can accelerate pericyte maturation in CNV lesions (Figure 5E, F). No significant differences in the levels of PDGFb, PDGFRb and TGFβ1 were observed between FasL^ΔCS/ΔCS^ and WT mice (Figure 5D, E, and F). Taken together, these data indicate that increased expression of PDGFRb and TGFβ1 in the retinas of FasL^ΔCS/WT^ mice coincides with increased pericyte recruitment/maturation in CNV, resulting in accelerated vessel stabilization and inhibition of vascular leakage.

### VEGF, PEDF and vascular normalization

**Fig. 6:**
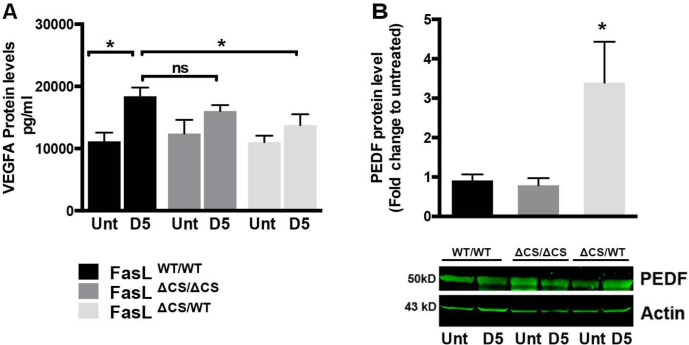
Proangiogenic and antiangiogenic protein expression analysis. (A) ELISA was performed to quantitate protein levels of VEGFA in posterior eyecups taken at day 5 post laser. Data are shown as mean pg/ml of VEGFA protein ± SEM per posterior eyecup, N=4–6 mice per group from 2 independent experiments. (B) Western blot was performed to quantitate protein levels of PEDF. Data are shown as fold change from untreated (Day 0) ± SEM. N=4–6 mice per group from 2 independent experiments. P-values refer to one way ANOVA and Tukey’s multiple comparisons analysis, *P<0.05.

Vascular leakage is regulated, in part, by the ratio of vascular endothelial growth factor (VEGF) and pigment-epithelium derived factor (PEDF) in the retina [44–46]. VEGFA in particular is one of the key factors controlling endothelial cell function in the eye [47] and the importance of VEGFA in vascular leakage is underscored by the clinical success of VEGFA neutralizing antibodies (bevacizumb, ranibizumab) in the treatment of “wet” AMD [48–51]. PEDF is a potent anti-angiogenic factor that has been shown to inhibit VEGF-induced vascular permeability both in vitro and in vivo [45, 46]. To determine whether changes in the levels of VEGFA / PEDF coincided with the reduced vascular leakage in FasL^ΔCS/WT^ mice, we examined protein levels for VEGFA and PEDF using eyecups (retina, RPE, choroid) each containing 4 CNV lesions obtained from WT, FasL^ΔCS/WT^, and FasL^ΔCS/ΔCS^ mice at 5 days post laser treatment (Figure 6A and B). Eyecups obtained from untreated mice (no laser treatment) were used as negative controls. The protein level of VEGF was significantly elevated in laser treated WT mice, but not in laser treated FasL^ΔCS/WT^ or FasL^ΔCS/ΔCS^ mice (Figure 6A). By contrast, PEDF protein levels were significantly increased in laser treated FasL^ΔCS/WT^ mice but not WT or FasL^ΔCS/ΔCS^ mice (Figure 6B). No significant differences in VEGFA and PEDF protein levels were observed between WT and FasL^ΔCS/ΔCS^ mice (Figure 6A and B). These data indicate that reduced vascular leakage within CNV lesions of FasL^ΔCS/WT^ mice coincided with decreased levels of VEGFA and increased expression of PEDF, which is known to inhibit VEGF-induced vascular permeability and thereby promote vessel normalization.

### Fas-FasL signaling in myeloid cells dictate neovascularization

**Fig. 7:**
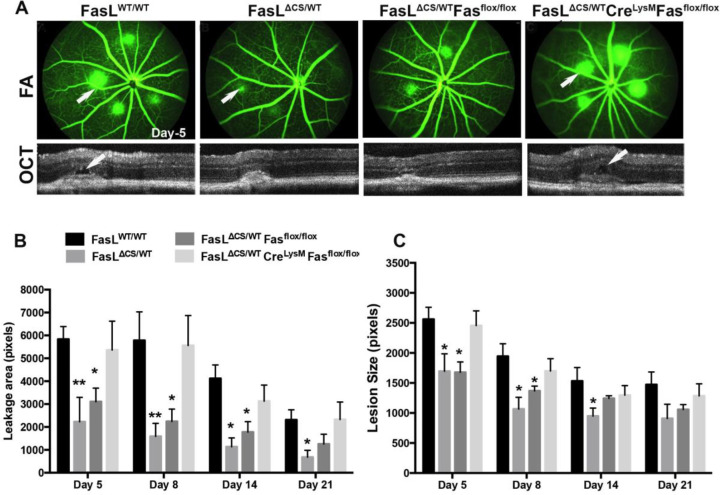
Fas expression on myeloid cells mediates protective phenotype in FasL ΔCS/WT mice. (A) Representative FA and OCT images acquired at day 5 post laser injury for FasL^WT/WT^, FasL^ΔCS/WT^, FasL^ΔCS/WT^.Fas^FLOX/FLOX^, and FasL^ΔCS/WT^.CreLysM, Fas^FLOX/FLOX^ mice (white arrows show area of leakage in FA and OCT). (B) Quantification of the area of leakage by FA at 5, 8, 14, and 21 days post laser injury. Shown is the mean area in pixels ± SEM. (C) Quantification of CNV size by SD-OCT at Day 5, 8, 14, and 21 post laser injury. Shown is the mean area in pixels ± SEM. FasL^WT/WT^ (N=8), FasL^ΔCS/WT^ (N=4), FasL^ΔCS/WT^.Fas^FLOX/FLOX^(N=4), and FasL^ΔCS/WT^.CreLysM, Fas^FLOX/FLOX^ (N=10). P-values refer to 2-way ANOVA and Bonferroni’s multiple comparisons analysis. *P,0.05, **P<0.01.

Vascular normalization is an emerging strategy to enhance cancer immunotherapy and several cancer studies indicate that vascular normalization is mediated in part by tumor-associated macrophages [52–56]. Moreover, work by Apte et al., demonstrate that macrophages are protective against the development of CNV in the eye[57]. Therefore, we asked whether Fas expressing myeloid cells mediate the protective phenotype observed in heterozygous FasL^ΔCS/WT^ mice. To address this question, heterozygous FasL^ΔCS/WT^ mice that conditionally lack Fas only in the myeloid cell compartment (FasL^ΔCS/+^ Fas^flox/flox^ Cre^LysM^) were generated. PCR confirmed deletion of the Fas gene specifically within CD11b^+^-sorted splenic myeloid cells from FasL^ΔCS/+^ Fas^flox/flox^ Cre^LysM^ mice (supplemental figure 2). To determine whether Fas expressing myeloid cells mediated vascular normalization in heterozygous FasL^ΔCS/WT^ mice, we used a 532 nm laser to induce CNV in FasL^ΔCS/+^ Fas^flox/flox^ Cre^LysM^ mice that lack Fas in the myeloid compartment and compared to FasL^WT/WT^ and FasL^ΔCS/+^ mice that do express Fas in the myeloid compartment. As a control for the effect of Fas^flox/flox^, we used FasL^ΔCS/+^ Fas^flox/flox^ mice that did not express LysMCre. Following laser injury, we determined the size of CNV by SD-OCT and the area of vascular leakage by fluorescein angiography on days 5–21 post laser treatment. Consistent with Figure 3, the area of leakage and lesion size was significantly reduced in FasL^ΔCS/+^ mice when compared FasL^WT/WT^ mice (Figure 7). In the absence of Cre, the FasL^ΔCS/+^ Fas^flox/flox^ mice showed no significant difference in the area of leakage and lesion size when compared to FasL^ΔCS/+^ mice. However, in absence of Fas expressing myeloid cells, FasL^ΔCS/+^ Fas^flox/flox^ Cre^Lysm^ mice presented with significant vascular leakage and CNV lesions equal to those observed in WT controls. Taken together, these data indicate Fas expressing myeloid cells mediate the vascular normalization observed in heterozygous FasL^ΔCS/WT^ mice.

## Discussion

Choroidal neovascularization is the hallmark of “wet” AMD where abnormal choroidal vessels infiltrate into the outer retina of the macula. These new blood vessels often bleed and leak fluid, causing retinal swelling and detachment with subsequent loss of vision. Fas ligand (FasL) is constitutively expressed in the eye and has been implicated in the inhibition of neovascularization in several *in vivo* models of neovascularization (suture-induced, hyperoxia-induced, and laser-induced), where increased neovascularization was observed in FasL deficient mice (gld/gld) [15, 58, 59]. In all cases, it was concluded that FasL controlled neovascularization by inducing apoptosis of infiltrating Fas+ endothelial cells. Moreover, in 2006, Semkova et al. used a transgenic model to demonstrate that specific overexpression of FasL on retinal pigment epithelial (RPE) cells prevented laser-induced CNV [16] thereby identifying the Fas-FasL pathway as a potential therapeutic target in the treatment of CNV.

FasL exists as both a membrane-bound (mFasL) and soluble (sFasL) protein and it has been demonstrated by a variety of laboratories studying FasL in non-ocular tissues and by our laboratory studying FasL within the eye that mFasL is pro-apoptotic and pro-inflammatory, while sFasL is non-apoptotic and anti-inflammatory add more refs [27–29, 35, 60, 61]. Therefore, we predicted that laser-induced CNV in mice that overexpress the membrane form of FasL and cannot produce soluble FasL (FasL^ΔCS/ΔCS^ mice) would result in a dramatic reduction in CNV lesion size and vascular leakage due to increased mFasL mediated apoptosis of infiltrating choroidal endothelial cells. By contrast, we observed no inhibition of invading choroidal endothelial cells into the retina of FasL^ΔCS/ΔCS^ mice following laser treatment and there was no significant difference in either lesion size or area of leakage between WT FasL^WT/WT^ mice and homozygous FasL^ΔCS/ΔCS^ mice. Moreover, the CNV lesions in WT and FasL^ΔCS/ΔCS^ mice contained disorganized, immature, and leaky vessels, characterized by poor pericyte coverage and lack of proper tight junctions between endothelial cells, similar to the sprouting microvessels found in patients with neovascular AMD that are immature, dilated, and lack organization or hierarchy. These data indicate that in the absence of sFasL, mFasL alone is unable to inhibit the development of laser induced CNV.

Most importantly, however, our experiments revealed a very unexpected and unique vascular phenotype in heterozygous FasL^ΔCS/WT^ mice, which displayed significant reduction in the lesion size and area of vascular leakage, as compared to WT FasL^WT/WT^ mice. We expected these smaller lesions in heterozygous FasL^ΔCS/WT^ mice would coincide with a reduced ingrowth of choroidal vessels into the retina. However, to our surprise, we observed the opposite result, CNV lesions in heterozygous FasL^ΔCS/WT^ mice contained numerous choroidal vessels that were highly organized, well perfused, and displayed normal pericyte coverage, all hallmark of mature vessels [53, 62]. These results suggest that the prior results obtained with doxycycline treatment resulted from only partial blockade of FasL cleavage. More importantly, the data clearly demonstrate that the extent of FasL cleavage has a dramatic impact on how FasL regulates CNV development.

In physiologic angiogenesis, newly formed vessels mature quickly, stabilize, and cease proliferation. However, in pathologic angiogenesis, such as in neovascular AMD, an imbalance of anti- and pro-angiogenic factors results in the uncontrolled growth of neovessels that are abnormal and leaky, and under constant reconstruction [63]. The high VEGF (pro-angiogenic) to PEDF (anti-angiogenic) ratio expressed in the eyes of WT mice following laser injury, coincided with CNV lesions characterized by leaky immature vessels that lack proper pericyte coverage, resulting in significant vascular leakage. By contrast, the significant increase in PEDF expression detected in the heterozygous FasL^ΔCS/WT^ mice coincided with the formation of mature, non-leaky vessels.

Studies of tumor vascular development demonstrate that it is possible to prevent pathologic angiogenesis by restoring the angiogenic balance thereby promoting vessel maturation and normalization [64–66]. The “Vascular Normalization Hypothesis” states that direct or indirect anti-angiogenic therapy, aimed at inhibiting pro-angiogenic factors and upregulating anti-angiogenic factors restores the angiogenic balance [64, 66]. As a result, vessels mature with increased pericyte coverage, proper endothelial cell tight junctions, normal perfusion, and reduced vascular leakage [42, 66]. The data presented herein suggest that the interplay between regulation of FasL cleavage and increased expression of PDGFRb, TGFβ1 and PEDF in the heterozygous FasL^ΔCS/WT^ mice restored angiogenic balance and achieved normalization by accelerating neovessel maturation.

We have also shown that deletion of the Fas receptor on myeloid cells using an intercross of LysM-Cre and floxed-Fas strains completely eliminates the protective activity of FasL in the FasL^ΔCS/WT^ mice. These data point to the myeloid subset as the critical Fas target and potential source of factor(s) that stimulate vascular normalization in the FasL^ΔCS/WT^ mice. We and others have previously shown that FasL can induce the production of a variety of cytokines in Fas^+^ macrophages that are relatively insensitive to FasL-mediated apoptosis [67–69]. Moreover, FasR engagement has been shown to drive M1-polarization in macrophages [70] and M1-macrophages are associated with vessel normalization[54, 71, 72]. However, it is not immediately clear why FasL^ΔCS/ΔCS^ myeloid cells would not serve a similar function. One possible explanation is a Goldilocks effect – the stronger FasL signal in FasL^ΔCS/ΔCS^ mice may lead to the demise of the Fas^+^ myeloid cells that in FasL^ΔCS/WT^ mice produce the factors that promote neovessel maturation.

In conclusion, while the emergence of anti-VEGF antibodies has revolutionized the treatment for CNV, restoring and preserving vision in many patients, the repeated intravitreal injections combined with the loss of efficacy over time and development of outer retinal atrophy has brought into question the long-term benefits of anti-VEGF therapy [73]. Our results indicate that FasL-mediated inhibition of vascular leakage in a mouse model of AMD results from a *non*apoptotic pathway that promotes accelerated neovessel maturation mediated by infiltrating myeloid cells and this effect is highly dependent on the extent of FasL cleavage as only heterozygous FasL^ΔCS/WT^ mice, and not homozygous FasL^ΔCS/ΔCS^ mice, exhibited reduced CNV. Future studies will be focused on elucidating the mechanism(s) by which sFasL and mFasL drive macrophage polarization and regulate neovessel maturation.

## Materials and methods

### Animals.

All animal experiments were approved by the Institutional Animal Care and Use Committee at Schepens Eye Research Institute, Boston, MA, and were performed under the guidelines of the Association for Research in Vision and Ophthalmology Resolution on the Use of Animals in Research. All mice were housed in an AAALAC approved animal facility at Schepens Eye Research Institute, given standard mouse chow and water ad libitum, and maintained on a 12 hr light/dark cycle. Male and female littermates (FasL ^WT/WT^, FasL^ΔCS/ΔCS^, FasL^ΔCS/WT^) at 10–12 weeks old were used in this study. The FasL^ΔCS/ΔCS^ mice were generated by a knock-in strategy described in a previously published manuscript [35]. The founder mice were backcrossed for 10 generations onto the C57BL/6J background to generate the FasL ^WT/WT^, FasL^ΔCS/ΔCS^, FasL^ΔCS/WT^ mice used in the studies described herein. To generate heterozygous FasL^ΔCS/WT^ mice that conditionally lack Fas only in the myeloid cell compartment, FasL^ΔCS/ΔCS^ mice were first crossed with C57BL/6 mice homozygous for the loxP flanked Fas allele (Fas^flox/flox^, The Jacksojn Laboratory) to generate mice that were homozygous for the non-cleavable ΔCS FasL allele and homozygous for the loxP flanked Fas allele and (FasL^ΔCS/ΔCS^Fas^flox/flox^). The FasL^ΔCS/ΔCS^Fas^floox/flox^ mice were then crossed with mice expressing Cre under control of murine lysozyme M gene promoter (Cre^Lysm^; The Jackson Laboratory), thereby generating FasL^ΔCS/WT^Cre^Lysm^ Fas^flox/flox^ mice. All mice were anesthetized (for laser photocoagulation, SD-OCT, fluorescein angiography, and vascular perfusion) by intraperitoneal (IP) injection of ketamine (100mg/kg body weight) and Xylazine (20mg/kg body weight). Mice were euthanized (for tissue harvest) by CO_2_ inhalation followed by cervical dislocation.

### Laser photocoagulation.

Laser photocoagulation was performed using a 532-nm laser (Meridian) attached to the Micron III fundus camera (Phoenix Research Labs, Pleasanton, CA, USA). Following anesthesia, the pupils were dilated with topical application of 1% tropicamide. The fundus imager was used to focus the laser beam on the junction of the RPE/choroid. Four lesions were created using ~100 mW actual power for a duration of 50 ms at the 3, 6, 9, and 12’0 clock meridians centered on the optic nerve and located two-or three disc diameters from the optic nerve (Figure 2, Supplemental figure 1).

### Spectral Domain Optical coherence tomography (SD-OCT).

The size of CNV lesions was determined using a SD-OCT system (Bioptigen, Durham, NC). Pupils of anesthetized mice were dilated with topical application of 1% tropicamide. Genteal (Novartis) was applied to hydrate the ocular surface and maintain corneal clarity. Volume analysis centered on the optic nerve head was performed, using 100 horizontal, raster, and consecutive B-scan lines, each one composed by 1200 A- scans. In Vivo Vue 2.0 software was used to acquire images. Quantification of lesion size by OCT was performed using ImageJ software from NIH as described previously [36]. Briefly, OCT images were taken through the center of each lesion, defined as the midline passing through the area of the RPE-Bruch’s membrane rupture. The lesion size was calculated by outlining the entire area occupied by lesion and leakage using ImageJ software.

### Fundus imaging and fluorescein angiography (FA).

Fundus imaging was performed using the MICRON III camera (Phoenix Laboratories, CA, USA). Pupils of anesthetized mice were dilated with topical application of 1% tropicamide. Genteal (Novartis) was applied to maintain hydration of the ocular surface. Images were acquired using brightfield or GFP filters (excitation, 482 nm, emission, 536 nm). FA was performed following ip injection of 1% fluorescein (HUB pharmaceuticals, LLC, Rancho Cucamonga, CA) at a dose of 0.2ml/25 g body weight. FA images were taken at three different time points: 1–2 mins, 4–5 mins and 9–10 mins post ip-injection of fluorescein. The final images taken at 9–10 mins were turned into black and white images; and using contrast, the total white area was measured using ImageJ software and represented the total area of vascular leakage (Figure 2, Supplemental figure 1). Lesions were also graded using a previously established grading system [37]. Grade 0 (not leaky) faint hyperfluorescence or mottled fluorescence, no leakage; Grade 1 (questionable leakage) hyperflourescent lesion without progressive increase in size or intensity; Grade 2A (leaky) hyperfluorescence increasing in intensity but not in size; Grade 2B (Clinically significant leakage) hyperfluorescence increasing in intensity and size.

### FITC-Dextran Perfusion.

At the time point for euthanasia anesthetized mice were perfused through the left ventricle with 10 ml of 1xPBS followed by 7 ml 5 mg/ml FITC-dextran (Mol. Wt. 2 × 10^6^; Sigma, St. Louis, MO). Following perfusion, the eyes were enucleated and processed for immunohistochemistry.

### Immunohistochemistry.

Posterior eyecups (retina, RPE, choroid and sclera) were fixed in 4% paraformaldehyde (PFA) for 2 hours and embedded in OCT media for frozen sections or placed in 1xPBS overnight for choroidal whole-mount preparations. Retinal frozen sections or choroidal whole mounts were then incubated in blocking solution (0.5% BSA, 0.2% Triton in 1xPBS) for 1 hour at RT and incubated overnight with the primary antibody. Collagen IV (Abcam-ab19808) was used at 1:100, NG2 (Abcam-ab83178) at 1:100, cd31 (BD Pharmingen cat #550274) at 1:50 concentrations. AlexaFlour secondary antibodies were used at 1:500. Confocal images were acquired with a Leica confocal microscope and processed using the SP6 software. Collagen IV staining was used to assess total lesion size and FITC-dextran perfusion was used to assess total area of perfused vessels. The % area of fluorescence per lesion was measured using ImageJ software

### Western Blot and ELISA.

Eyes were enucleated and placed in ice-cold 1 x PBS with protease inhibitor cocktail (pH 7.5; Roche Chemicals, Basel, Switzerland). The anterior segment, including the cornea, iris, ciliary body, and lens were removed. The posterior eyecups (2 eye cups per sample) consisting of the retina, choroid, and RPE were homogenized using a pestle pellet homogenizer in 100ul RIPA buffer. The buffer was then collected, pooled and centrifuged at 13,000 rpm. Protein concentration was then determined using the Nanodrop 2000c (Thermo Scientific) and supernatants were used for Western Blot and ELISA. Invitrogen NuPage LDS sample buffer were used for loading in a 12% gel for Western Blot analysis. Transfer was performed using the iBlot dry transfer equipment from Invitrogen. PEDF antibody (Millipore cat # 07–280) was used at 1:100 concentrations, actin antibody 1:50 (sc1615, Santa Cruz). LI-COR secondary antibodies were used for detection according to manufacturer’s protocol. Odyssey CLx Infrared imaging system (LI-COR Biosciences, USA) was used to visualize the bands. Mouse VEGF ELISA kit from Sigma (cat# RAB0509–1KT) was used to quantify VEGF levels according to the manufacturer’s protocol.

### Quantitative Real time PCR.

Eyes were enucleated and RNA was isolated from posterior eyecups (retina, choroid, and sclera) using Qiagen RNeasy mini kit (Cat # 74104) according to the manufacturer’s protocol. RNA was treated with DNAse (Cat # AM222, Invitrogen) to ensure no contamination of genomic DNA. 500ng of RNA was used to prepare cDNA using the iScript cDNA synthesis kit from Bio-Rad (Cat # 170–8890) according to the manufacturer’s protocol. cDNA was treated with RNaseH (18021–014, Invitrogen) to ensure absence of single stranded RNA. Quantitative real time PCR was performed using the master mix from Invitrogen (Cat # 4472903) using the manufacturer’s protocol in 10μl total volume in duplicates. Relative expression to housekeeping gene β-actin was quantified using the formula: Relative expression =10,000 × 1/2^(Avg. Gene cT- Avg. β-actin cT). Fold changes were calculated with respect to untreated control eyes. The primers used include: β- actin- F-5’ AAATCGTGCGTGACATCAAA-3’R-5’ AAGGAAGGCTGGAAAAGAGC 3’, TGFβ1-F- 5’ GCTGCGCTTGCAGAGATTAAA 3’R-5’-TTGCTGTACTGTGTGTCCAG 3’, PDGFb- F-5’-ACTCCATCCGCTCCTTTGAT- 3’R-5’-GTCTTGCACTCGGCGATTA- 3’ and PDGFbR- F-5’ CAACCGTACCTTGGGTGACT 3’R-5’-GAGAGCTGGACCTCATCGTC 3’.

### Statistical analyses.

Graph pad prism 6 (La Jolla, CA, USA) was used for statistics. Statistics were performed as indicated in each figure legend using two-way ANOVA and Bonferroni’s post-hoc comparisons or one-way ANOVA and Tukey’s post-hoc comparisons. Significance was defined at a P value <0.05.

## Data Availability

The data presented in this article will be shared on reasonable request to the corresponding author.
